# Effect of Fe nanoparticle on growth and glycolipid biosurfactant production under solid state culture by marine *Nocardiopsis* sp. MSA13A

**DOI:** 10.1186/1472-6750-14-48

**Published:** 2014-05-21

**Authors:** George Seghal Kiran, Lipton Anuj Nishanth, Sethu Priyadharshini, Kumar Anitha, Joseph Selvin

**Affiliations:** 1 Department of Food Science and Technology, Pondicherry University, Puducherry 605014, India; 2 Department of Microbiology, School of Life Sciences, Pondicherry University, Puducherry 605014, India

**Keywords:** Fe nanoparticles, Biosurfactant, Actinobacterium, Solid state fermentation, Nanoparticle-microbial interaction

## Abstract

**Background:**

Iron is an essential element in several pathways of microbial metabolism, and therefore low iron toxicity is expected on the usage of Fe nanoparticles (NPs). This study aims to determine the effect of Fe NPs on biosurfactant production by marine actinobacterium *Nocardiopsis* sp. MSA13A under solid state culture. Foam method was used in the production of Fe NPs which were long and fiber shaped in nature.

**Results:**

The SEM observation showed non toxic nature of Fe NPs as no change in the morphology of the filamentous structure of *Nocardiopsis* MSA13A. The production of biosurfactant by *Nocardiopsis* MSA13A under solid state culture supplemented with Fe NPs increased to 80% over control. The biosurfactant produced by *Nocardiopsis* MSA13A was characterized as glycolipid derivative which effectively disrupted the pre-formed biofilm of *Vibrio* pathogen.

**Conclusion:**

The use of metal NPs as supplement would reduce the impact of non-metallic ions of the metal salts in a fermentation process. This would ultimately useful to achieve greener production process for biosurfactants. The present results are first report on the optimization of biosurfactant production under SSC using Fe NPs.

## Background

Production economy is a greater challenge in industrial production of biosurfactants. Successful production depends on the development of low-cost production processes and readily available raw materials which could reduce the cost of production process substantially [1]. Development of economical engineering processes and the use of low cost feedstock for microorganism growth and surfactant production could reduce the production cost [2]. It is a well-known fact that microorganisms are immensely affected by conditions that prevail surrounding them. Variations in carbon and nitrogen sources along with its stabilization at different pH and temperature have resulted in increased yield of biosurfactants. Metal supplementation was reported to be one of the critical factors required for enhanced production of biosurfactants [3]. Among the metal ions, Fe is the key microelement for biosurfactant production in several microorganisms [4]. Based on our previous bioprocess optimization for production of biosurfactants by marine actinobacteria [5,6], it was established that metal ions such as FeCl_3_, CuSO_4_, MnCl_2_, and MgSO_4_ had significantly influenced biosurfactant production. Among the metal ions screened, FeCl_3_ was most appropriate metal precursor for the production of biosurfactant by marine actinobacteria followed by CuSO_4_. Makkar and Cameotra [7] reported that the biosurfactant production increased considerably (almost double) by the addition of metal supplements. Considering the significant role of metal ion on biosurfactant production, the present study was initiated to evaluate the effect of metal (Fe^3+^) NPs on biosurfactant production.

Nanoparticles (NPs) have been hypothesized to be influence microbial production, growth and survival. Previous reports evidenced the effect of different NPs on the growth and secondary metabolite production in various microorganisms [8,9]. But reports revealed that NPs like Ag, Au and oxides of Al, Ti, Si and Zn have detrimental effect on the cells of microorganisms [10]. Fe was considered as an essential element with many other novel properties, such as enhanced surface-to-volume ratio, super-paramagnetism and inherent biocompatibility [11,12]. Because of using Fe atom in several pathways of metabolism, low iron toxicity is expected on the usage of Fe NPs [13,14]. In this background, the present study aims to determine the effect of Fe NPs on biosurfactant production by marine actinobacterium under solid state culture (SSC).

## Results and discussion

### Isolation and identification of biosurfactant producer MSA13A

This report is an outcome of bioprospecting of actinobacteria associated with marine sponge *Dendilla nigra*. Among the 57 stable isolates (MSA01-MSA057), the strains MSA04, MSA10, MSA13A, MSA19 and MSA21 were considered as potential biosurfactant producers. In the present study, we report the biosurfactant production potential of MSA13A. The results of screening tests performed on MSA13A include hemolytic activity (6 mm), oil displacement (10 mm), lipase activity (72 U/mg), positive drop collapsing test and emulsification activity (23%). The isolate hydrolyzed starch, gelatin, chitin, cellulose and tributyrin. The 16S rRNA sequence of the isolate MSA13A was further analyzed using NCBI BLASTn tool with a query to limit the search for closest biosurfactant producing relatives. Representatives of maximum homologous sequences from the search were used for the construction of phylogenetic tree using UPGMA algorithm. The isolate MSA13A showed clustering with prominent biosurfactant producers *Bacillus* and *Pseudomonas*. These findings revealed that the isolate MSA13A was an actinobacterial strain producing extracellular biosurfactants. Based on the homology and similarity, the isolate was classified as *Nocardiopsis* sp. MSA13A. Sponge-associated actinobacteria have been reported to be prolific source of secondary metabolite producers [15,16] and suggested to attribute largely to the chemical defense mechanisms of their host sponges against predators with biologically active compounds (repellents) and biofouling ([17] and references therein).

### Interaction of NPs with actinobacterium

**Figure 1:**
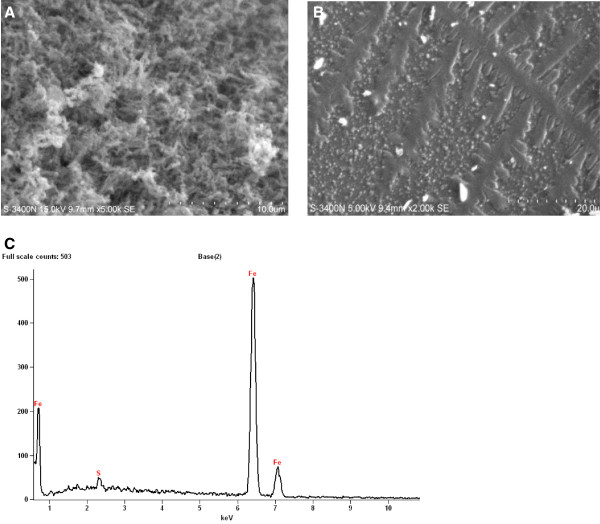
**SEM observation of (A) fiber-shaped Fe nanoparticles (diameter nano size) synthesized using foam method (B) *Nocardiopsis *** **morphology after 4 h interaction with 10 mg/L Fe NPs (C) EDS analysis of freshly synthesized Fe NPs showing high percentage of iron element.**

Foam method was used in the production of Fe NPs which were long and fiber shaped in nature (Figure 1A). The diameter of the synthesized Fe NPs was about 40 nm and they were around 1 μm in length. The SEM results showed fibrous shape of the Fe NPs along with their approximate size. EDS analysis evidenced iron to be the metal in Fe NPs synthesized (Figure 1C). Absence of oxygen indicated that the Fe NPs had not undergone oxidation even though mild exposure to air during the synthesis process. UV–vis spectroscopy of Fe NPs showed an absorption peak at 230 nm (data not shown). Fe NPs obviously show peaks at 216 nm and 268 nm in distilled water [18]. The strong absorbance obtained at a wavelength <250 nm indicated that the size of the Fe NPs synthesized was < 40 nm. It was observed that the highest densities of colonies were appeared on the plate with 10 mg/L of Fe nanoparticle. No significant change was observed between fresh and one week old Fe NPs. Growth rate of *Nocardiopsis* MSA13A was induced significantly by Fe NPs at 10 mg/L. The effect of Fe NPs at 100 mg/L and lower than 10 mg/L showed least or no effect on the growth of *Nocardiopsis* MSA13A. The reduction in cell density on addition of high concentration of Fe NPs was probably due to aggregation in the medium leading to damage of the growing cell [19-21]. The present study evidenced Fe NPs was non-toxic to actinobacterium whereas Cu NPs were reported to be toxic to bacterial cells [22]. Little change was observed with one week old Fe NPs indicating the variation in toxicity of the NPs probably due to their oxidation but still the overall effect had remained the same. To avoid oxidative effect on Fe NPs, the present study was carried out with freshly prepared Fe NPs. The Fe NPs positively interacted with actinobacterium which was evident from the intact filamentous morphology of *Nocardiopsis* MSA13A treated with 10 mg/L of Fe NPs for 4 h (Figure 1C).

### Influence of Fe NPs on biosurfactant production

**Figure 2:**
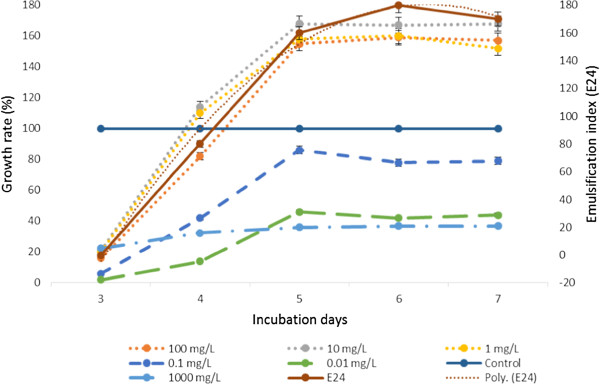
**Effect of Fe NPs on the growth rate and biosurfactant production by *Nocardiopsis *MSA13A.** Lag period was fall on 0 to 2 d which is not shown in the Figure. The growth rate was presented in terms of percent increase over the control. Growth rate was derived from colony plate count and OD values. 10 mg/L Fe NPs induced growth rate of *Nocardiopsis* MSA13A to the maximum of 67% over control. The same Fe NPs concentration increased to 80% biosurfactant production over control.

The production of biosurfactant by *Nocardiopsis* MSA13A was increased to 80% over control (without NPs) at 10 mg/L Fe NPs (Figure 2). It was recorded that the production of biosurfactant decreased at higher concentration of Fe NPs. The initial increase in production was due to the effect of Fe nanoparticle providing more nutrition to the organism by activation of the medium [14]. Electron donating nature of the Fe^2+^ ion has been studied previously which satiates the demand of the organism for electron [4]. Ehrlich [23] reported that some bacteria can acquire energy for growth from oxidation of Fe^2+^ to Fe^3+^. Fe is an important enzyme activator, specifically of isocitrate lyase, an enzyme involved in cell growth on hydrophobic substrates. This enzyme is essential for cell to modulate metabolic pathway of acetyl-CoA and convert it into a C4 unit during biosurfactant synthesis [24]. The microorganisms have developed a variety of strategies for acquiring iron while simultaneously protecting them from the potential toxicity of iron [25]. The main strategies used by bacteria and fungi to acquire iron include producing and utilizing siderophores.

### Optimization of biosurfactant production under SSC

**Figure 3:**
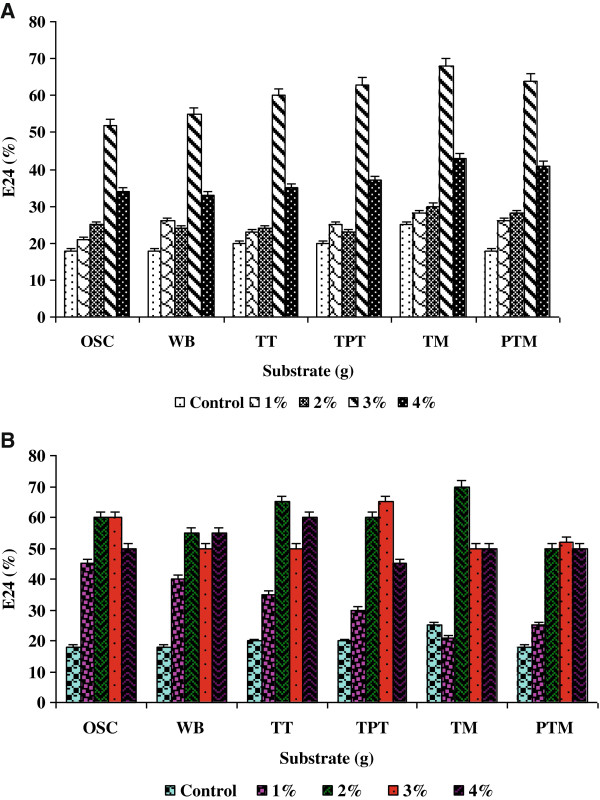
**Effect of carbon and nitrogen sources on biosurfactant production. A)** Effect of increasing concentration of optimized carbon source, glucose on the production of biosurfactant **B)** Effect of increasing concentration of optimized nitrogen source, yeast extract on the production of biosurfactant by *Nocardiopsis* MSA13A.

The production of biosurfactant by *Nocardiopsis* MSA13A was increased substantially with treated molasses (distillery waste) as substrate (E_24_ 35%) followed by tannery pretreated sludge (20%), pre-treated molasses (20%), tannery treated sludge (20%) and wheat bran (18%). Enhanced biosurfactant production by *Nocardiopsis* MSA13A under SSC was achieved with glucose as carbon source, yeast extract as nitrogen source, pH 7.0, 2% NaCl, and asparagine as amino acid. The production of biosurfactant by *Nocardiopsis* MSA13A was consistently increased between 30°C and 40°C. The influence of metal ions such as CuSO_4_, MnCl_2_, MgSO_4_ and FeCl_3_ on the production of biosurfactant by *Nocardiopsis* MSA13A was evaluated under SSC. Among the metal ions screened, FeCl_3_ was most essential metal ion for enhanced biosurfactant production by *Nocardiopsis* MSA13A followed by CuSO_4_. The strain showed maximum production on the substrate inoculated with 2.5 ml inoculum. The moisture content required for the maximum production of biosurfactant was >80%. Factors optimized under one-factor at a time experiments are given in supplementary figure. The strain *Nocardiopsis* MSA13A produced maximum biosurfactant on treated molasses with 3% glucose as carbon source and 2% yeast extract as nitrogen source (Figure 3A and B). The strain required a higher amount of carbon source compared to that of the nitrogen source concentration based on the effect of C/N ratio.

### Optimization of biosurfactant production using Response Surface Methodology (RSM)

Statistical optimization of biosurfactant production was carried out by Design-Expert software package (Stat-Ease, Inc., USA). The R^2^ value of 0.9406 which was closer to 1 shows the model to be stronger and it can better predict the response. The model was found to be significant with p < 0.0001 and insignificant lack of fit (Additional file 1). The behavior of the system is explained by the following quadratic model equation

$$\begin{array}{c} Y=40+10.67X_{1}-6.42X_{2}-11.42X_{3}-3.17X_{4}+16X_{1}^{2}\\ \;+7.88X_{2}^{2}+8.88X_{3}^{2}-16.25X_{4}^{2}+6.25X_{1}X_{2}\\ \;+0.75X_{1}X_{3}-4.5X_{1}X_{4}+2.5X_{2}X_{3}-3.0X_{2}X_{4}\\ \;-1.5X_{3}X_{4} \end{array}$$

### Validation of the model

**Figure 4:**
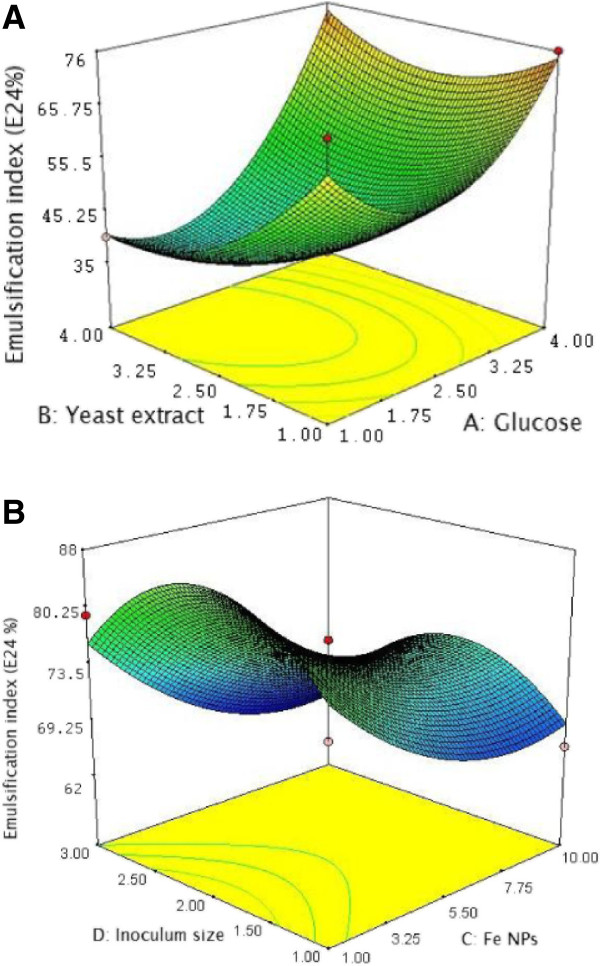
**Statistical optimization of biosurfactant production. A**. Contour plot of the interaction between the glucose (carbon source) and yeast extract (nitrogen source) on the production of biosurfactant in SSC by *Nocardiopsis* MSA13A. **B**. Contour plot of the interaction between the Fe NPs and inoculum size on the production of biosurfactant.

The predicted value of Y by the above explained quadratic model was found to be 72%. The experimental value obtained was 83%. The difference in the predicted and the experimental value can be attributed to Fe NPs. The Fe NPs might have induced the metabolic pathway of biosurfactant synthesis. It was found that the production was significantly influenced by the variables such as glucose, yeast extract, Fe NPs and inoculum size either interactively and/or independently. A significant interactive influence of primary control factors such as glucose and yeast extract was validated in the RSM design. The glucose and yeast extract interactively reached a central value to influence the production maxima over a stable area (Figure 4A). The Fe NPs and inoculum size interactively increase the production and the stable production was attained with the influence of both factors independently (Figure 4B). The present results are first report on the optimization of biosurfactant production using Fe NPs.

### Chemical characterization of biosurfactant

**Figure 5:**
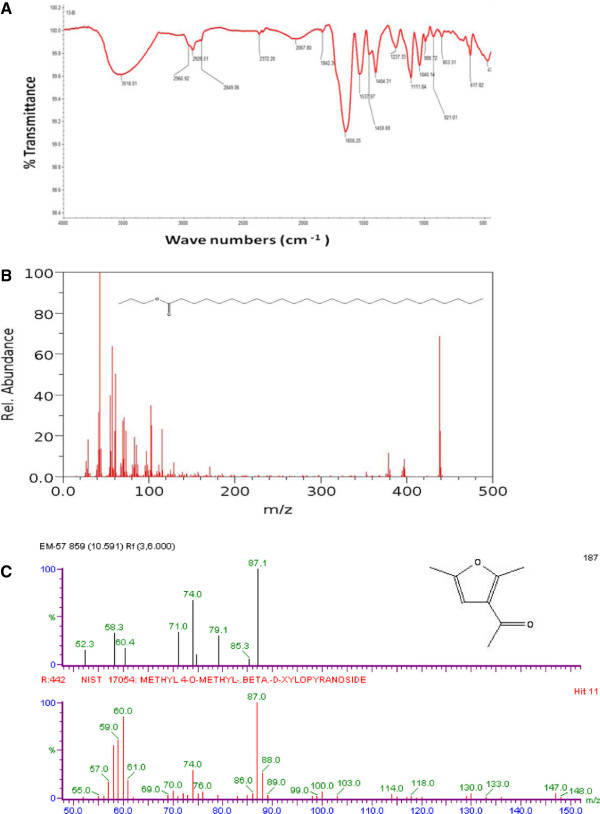
**Purification and chemical characterization of biosurfactant. A)** TLC results of carbohydrate and lipid fractions of biosurfactant. **B)** MS spectra of lipid moiety of glycolipid biosurfactant showing non-polar hydrocarbon chain (hexacosanoic acid, propyl ester). **C)** MS spectra of sugar moiety of glycolipid biosurfactant showing methyl-4- O-methyl-beta-D-xylopyranoside.

Based on the TLC analysis, carbohydrate and lipid fractions were separated. The FT-IR spectra (Figure 5A) and GC-MS data (Figure 5B and C) showed that the compound produced by *Nocardiopsis* MSA13A was a glycolipid with a hydrophobic non-polar hydrocarbon chain (hexacosanoic acid, propyl ester) and hydrophilic part of the compound being a sugar methyl-4- O-methyl-β-D-xylopyranoside. Retention times, relative intensities (%) and EIMS of the relevant peaks are as follows: Peak A: 10.591, 30, EIMS m/z (% rel. intensity), 87.1 (100), 79.1 (30), 76 (70), 71 (40), 60.4 (20), 58.3 (40) and 52.3 (15). Peak B: 18.058, 100, EIMS m/z (% rel. intensity), 420 (70), 105 (20), 100 (30), 60 (60), 40 (100), 20 (15). It was established that GC-MS was a standard technique in carbohydrate analysis [26] and based on the previous reports, the peaks obtained the present study was matched with methyl-4- O-methyl-β-D-xylopyranoside.

### Antibiofilm activity of biosurfactant

**Figure 6:**
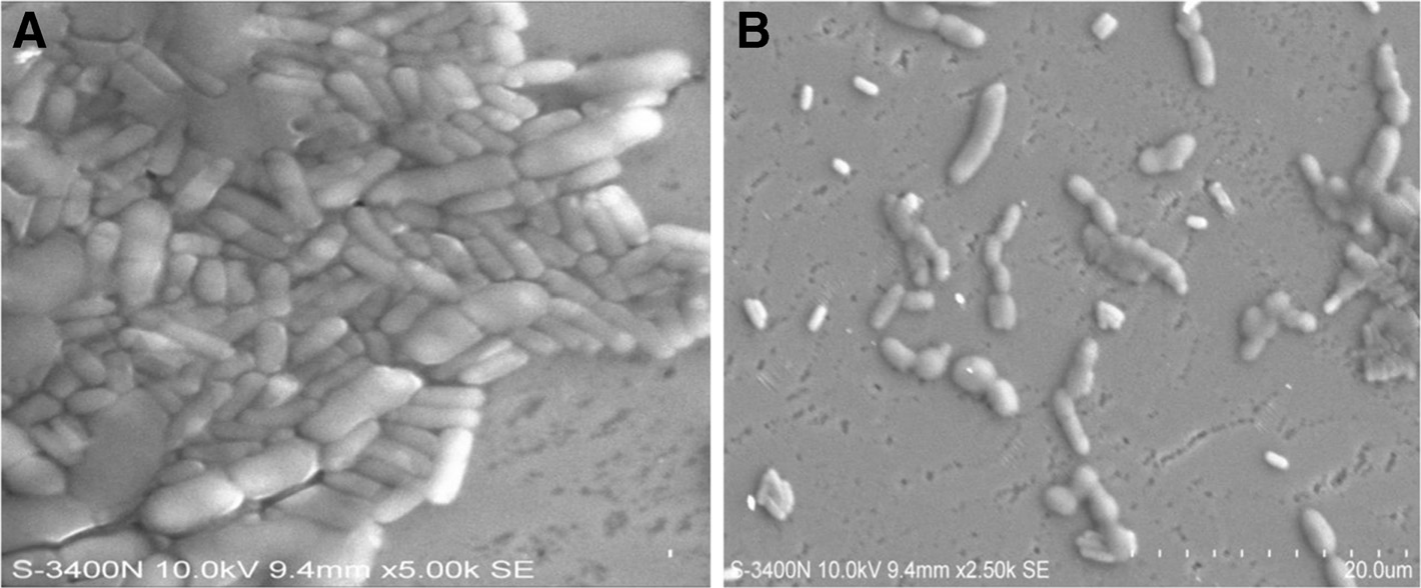
**Effect of biosurfactant on biofilm disruption. (A)** The cells were tightly bound by exopolysaccharies, shows biofilm **(B)** The effect of biosurfactant on the pre-formed (24 h) biofilm. The biofilm nature was disrupted and few microcolonies of *Vibrio alginolyticus* was remaining after the disruption of preformed biofilm by biosurfactant.

A prominent shrimp pathogen *Vibrio aliginolyticus* treated with 300 μg/ml of biosurfactant significantly disrupted the pre-formed biofilm (Figure 6). The biofilm disruption was indicated by the absence of visible film on the wall and bottom of the tube when the glass tube was incubated with biosurfactant (300 μg/ml) for 30 min at 28°C. Lower concentration was effective but not disrupted the biofilm completely. Vibriosis was by far the most significant factor of aquaculture loss in many countries. *Vibrio* is a serious pathogen of marine fish and invertebrates, particularly penaeid shrimp [27]. The finding of the present study brings out a new biosurfactant derivative from marine actinobacterium which could effectively disrupt pathogenic biofilms.

## Conclusion

Identification of bacterial responses provides vital information on the influence of NPs on microorganism. Due to their tiny size and stabilization [28], as well as high surface to volume ratio nanomaterials can easily influence the growth as well as secondary metabolite production of microorganisms, but this effect is not always negative in nature. In this study Fe NPs was used to enhance biosurfactant production by actinobacterium, since Fe is an essential nutrient for almost all microorganisms because it plays an important role in optimum cell growth as well as cofactor for a large number of enzymes, is a part of cytochromes and is required for many biochemical reaction, including respiration, photosynthetic transport, and DNA synthesis [29]. It has been hypothesized that the use of metal salt as supplements in the fermentation process would retain the non-metal salt ions such as Cl^-^ or SO_4_^2-^ as impurity in the medium. Therefore, the use of metal NPs as supplement would reduce residual effect of metal salts in the fermentation products. Based on the findings, the NPs could be used as inducer of metabolic pathway of actinobacterium for enhanced production of biosurfactant under SSC. The present study evidenced that the biosurfactant can be used to effectively disrupt and/or to prevent *Vibrio* sp. biofilms in shrimp aquaculture.

## Methods

### Isolation, screening and identification of marine actinobacterium

The collection and isolation of actinobacteria from the marine sponge *Dendrilla nigra* was performed as per the procedure of [30]. Briefly, 1 cm^3^ of sponge tissue was excised from the internal mesohyl area using a pair of sterile scissors. The excised portion was homogenized with phosphate buffered saline using a tissue homogenizer. The aliquot was placed on various isolation media including marine sponge agar [31] and standard media (HiMedia). The inoculated plates were incubated at 27°C for 14 days in dark. The incubation temperature was reduced to achieve a near environmental temperature (25°C) range prevailed at the site of sponge collection. The morphologically distinct colonies were reisolated and maintained on actinomycetes isolation agar (HiMedia) at 4°C. The isolates were screened for biosurfactant production using drop collapsing test, oil displacement test [32], lipase activity [33], and hemolytic activity [33]. Emulsification activity was performed according to Paraszkiewicz et al. [34]. All the assays were performed in triplicate with distilled water as control. The actinomycetes isolation agar was included as negative control in the screening as to determine the effect of medium on the emulsification index of the isolate. The producer strain MSA13A was identified morphologically and biochemically according to the method of Lechevalier [35] and the genomic DNA was obtained by the method of Ferrara et al. [36]. For the 16S rRNA sequencing the PCR analysis was performed as follows: Universal 16S rRNA eubacterial primer (5′-GAGTTTGATCCTGGCTCAG-3′; 5′-AGAAAGGAGGTGATCCAGCC-3′) was used for the amplification of 16S rRNA. The 16S rRNA gene sequence (FJ372669) obtained from the isolate MSA13A was compared with other bacterial sequences by using NCBI megaBLAST (http://blast.ncbi.nlm.nih.gov/Blast.cgi) for their pair wise identities. Phylogenetic tree was constructed in MEGA 4.0 version (http://www.megasoftware.net) using unweighted pair group method with arithmetic mean (UPGMA) algorithms (data not shown).

### Synthesis of Fe

Foam method was followed in the synthesis of Fe NPs chemically [14]. The NPs were synthesized in small batches of 0.27 g. Initially, 200 ml of FeSO_4._7H_2_O solution (6 g/L) was prepared along with 250 ml of the cationic surfactant CTAB (3.2 g/L). Both the solution was thoroughly mixed for 10 min on a magnetic stirrer. NaBH_4_ solution (22 g/L) was prepared in 15 mL of deionised water and mixed with FeSO_4._7H_2_O-CTAB solution for 15 min on a magnetic stirrer. On addition of NaBH_4_, the clear solution turned black in colour indicating the production of Fe NPs. The NPs were allowed to settle down for 15 min. The solution was flushed with acetone for six times and stored in acetone.

### Effect of NPs on growth of marine actinobacterium

Marine actinobacterium MSA13A (originally designated as actinobacterium MSA10) was cultured in actinomycetes broth (Himedia) with 1% glycerol and 2% NaCl at 30°C for 5 d to reach its exponential growth phase. NPs obtained by foam method were sonicated in phosphate buffered saline (PBS) for 30 min at 40 Hz until completely dispersed in the saline without any signs of agglomeration. The artificial dispersant was not used in the present study as they might interfere the interaction of NPs with biosurfactants. Freshly prepared as well as one week old Fe NPs dispersed in PBS to prepare broad range aliquots (0.01 to 1000 mg/L) and added into the culture of exponentially growing marine actinobacteriun MSA13A. Resultant actinobacterium-nanoparticle conjugate was incubated at 30°C for 24 h. The conjugate was diluted to 10^4^ fold and 10 μL each was plated on actinomycetes agar supplemented with 2% NaCl to determine growth rate by colony plate count method. After 5 d of incubation at 30°C, the number of colonies on triplicate plate was counted and calculated percent growth rate over control.

### Influence of NPs on biosurfactant production

The biosurfactant production was optimized in the preliminary phase under submerged fermentation (SmF) conditions. For the SmF, actinomycetes broth (Himedia) with trace elements - ZnSO_4_.7H_2_O-0.29 g, CaCl_2_.4H_2_O - 0.24 g, CuSO_4_.5H_2_O - 0.25 g, MnSO_4_.H_2_O-0.17 g/100 ml were used as production medium. 500 ml Erlenmeyer flasks containing 200 ml of medium was inoculated with actinobacterium and incubated at 30°C for 7 days. Based on the SmF conditions (data not shown), the production medium without trace elements were used for determining the effect of NPs on biosurfactant production.

Biosurfactant production medium without trace elements was enriched with a broad range of Fe NPs (0.01 to 1000 mg/L) and the growth rate of actinobacterium during the fermentation process was estimated based on OD at 600 nm (UV–vis spectrophotometer AU-2701). The emulsification index E_24_ was determined to find the effect of NPs on biodurfactant production. Briefly, olive oil was added to the cell free supernatant in a ratio of 1:1 and vortexed vigorously for 2 min. After 24 h of incubation, the height of the emulsified layer was measured and compared with the total height of the liquid layer and multiplied by 100 (E_24_). The direct effect of NPs on the biosurfactant activity was determined based on emulsification index. The cell free supernatant was extracted at least three times with chloroform–methanol (3:1, v/v), with 15 mL of this solvent mixture being used for each extraction. The organic phase was concentrated - at 40°C in a rotary vacuum evaporator (Yamato). Various concentrations of Fe NPs were mixed with biosurfactant extracted and incubated for 24 h. The E_24_ was then determined with olive oil emulsion as described above.

### SEM observation

Ethanol suspensions of immediately synthesized Fe NPs were vortexed and a thin, uniform smear was prepared on the surface of a cover slip. Sample prepared was then placed on carbon tape and was sputter-coated with carbon for SEM (S-3500 N Hitachi) observation. The interaction of Fe nanoparticle with the actinobacterium was observed under SEM. The exponentially growing actinobacterium were centrifuged at 1000 rpm for 10 min. The pellet obtained was resuspended in PBS containing the dispersed Fe nanoparticle and this actinobacterium-nanoparticle conjugate was incubated at room 30°C for 4 h. This conjugate was used to prepare thin and uniform smear on the surface of a cover slip. It was properly dehydrated and was placed into a carbon tape which was sputter-coated with carbon for SEM observation.

Energy-dispersive spectroscopy (EDS, Thermo, USA) was performed to determine the composition of the NPs. Along with freshly synthesized Fe NPs, one week old Fe NPs were also used as to find oxidative effect on the NPs. The spectral analysis was used to confirm the presence of elemental iron. It was also used to analyze the oxidized state of Fe NPs. Absorption spectrum of the synthesized Fe nanoparticle was measured by OD 500–900 nm scan in a UV–VIS spectrophotometer. Fe NPs were used without any dilution.

### Optimization of biosurfactant production under solid state culture (SSC)

For the development of SSC, the production substrate was developed using agro-industrial and industrial waste [37]. Based on the preliminary screening results, treated molasses (distillery waste), tannery pretreated sludge, pre-treated molasses, tannery treated sludge and wheat bran were selected for optimization experiments. The substrates were dried at 60°C in an oven prior to SSC formulation. The bioprocess was developed as per Kiran et al. [37]. Optimization of biosurfactant production was carried out by search one at a time experiments. Factors such as carbon and nitrogen sources, pH, temperature, amino acids, metal ions, inoculum size and salt concentration affecting the biosurfactant production were determined (Additional file 1). Subsequently response surface methods (RSM) were applied to analyze the interactions between the critical control factors [37,38]. In the RSM experiments, the ferric chloride was replaced with Fe NPs to determine the effect of NPs on biosurfactant production. The variables including glucose, yeast extract, Fe NPs and inoculum size that have effect on the production of biosurfactant were identified by the optimization experiments. Each independent variable was investigated at a high (+1) middle (0) and a low (-1) level. Runs of center points (control) were included in the matrix.

### Chemical characterization of biosurfactant

Extraction of glycolipids was performed with 100 mL of distilled water added to the SSC flasks and was agitated for 1 h at 200 rpm at 30°C on an orbital shaker. The suspension was filtered through cheesecloth, the excess liquid being squeezed out manually. This procedure was repeated three times. The extract was centrifuged for 10 min at 12,500 × g, and the supernatant was extracted at least three times with 15 mL each of chloroform–methanol (3:1, v/v) [39]. The organic phase was concentrated at reduced pressure at 40°C, giving rise to a crude extract containing the glycolipids. Glycolipids were quantified in terms of rhamnose using the phenol-sulfuric acid method [40] with sugar (rhamnose) as the standard. A control sample prepared from uncultured medium in order to check for interference from medium components. The presence of rhamnolipids was determined using correction factor [41]. To purify the surface active compound, the ethyl acetate extract was resolved through a column chromatography on reverse phase silica gel (230–400 mesh). Elution was performed with methanol from 65% to 100% at a flow rate of 0.5 ml/min at 30°C. An Agilent GC-MS system equipped with a fused silica capillary tube was used to analyze the components in this active fraction. The data was processed by GC-MSD Chemstation column condition was programmed as column oven temperature 150°C (4 min)–4°C/min, temperature of injection port 250°C and detector port 280°C. The peaks of the gas chromatography were subjected to mass-spectral analysis. The spectra were analyzed from the available library data. NIST MS search (version 2.0) (included with NIST’02 mass spectral library, Agilent p/n G1033A).

### Antibiofilm activity of biosurfactant

The biofilm strains were inoculated in LB broth and incubated at 37°C for 24 h. After incubation the cells were washed and resuspended in phosphate buffered saline (pH 7.2) to a turbidity equivalent to a 0.5 M McFarland standard. The 96- well U-bottomed microtitre plates were filled with 80 μl of LB broth, 10 μl of each cell suspension and 10 μl of each test concentration in triplicate. The control was set with newly developed biofilm. Triplicate wells each were set with biosurfactant concentrations such as 50, 100, 150, 200, 250 and 300 μg/ml. Plates were incubated on a platform shaker. After 24 h the planktonic cells and spent media were discarded, and adherent cells were rinsed with deionized water. Then the plates were allowed to air dry. The biofilms were stained by 200 μl of 0.4% crystal violet for 10 min. After staining the dye was discarded and the wells were rinsed twice with deionized water. The plates were air dried and 200 μl of dimethylsulfoxide was added to each well. The OD was determined at 595 nm in a microplate reader (data not shown). The SEM analysis was performed on preformed biofilm treated with effective concentration of biosurfactant as determined in the microplate assay. The biofilm disruption was evident from SEM observation.

## Competing interests

The authors declare that they have no competing interests.

## Authors’ contributions

JS designed, monitored and written the paper. ANL has performed Fe NPs synthesis and biofilm disruption part, GSK performed optimization part and AD and SP helped the lab experiments performed by GSK. All authors read and approved the final manuscript.

## Supplementary Material

Additional file 1: Table S1 Factors considered for the optimization of biosurfactant production under solid state culture (SSC). **Table S2.** ANOVA analysis of the optimization of production by *Nocardiopsis* MSA13A. **Figure S1.** Effect of various substrates including agro-industrial and industrial waste on the production of biosurfactant. The SSC was performed with basal medium 6 ml/250 ml flask, substrate 5 g/250 ml flask and 7 ml/5 g substrate. **Figure S2.** Effect of various carbon sources on the production of biosurfactant. **Figure S3.** Effect of various nitrogen sources on the production of biosurfactant. **Figure S4.** Effect of pH on the production of biosurfactant. **Figure S5.** Effect of tempertaure on the production of biosurfactant. **Figure S6.** Effect of incubation period on the production of biosurfactant. **Figure S7.** Effect of metal ions on the production of biosurfactant. **Figure S8.** GC-MS data of purified biosurfactant fraction. Click here for file
