# Supplementary material for: Effect of Fe nanoparticle on growth and glycolipid biosurfactant production under solid state culture by marine Nocardiopsis sp. MSA13A
Source: BMC Biotechnol. 2014 May 21;14:48. doi: 10.1186/1472-6750-14-48 (PMC4229807; doi:10.1186/1472-6750-14-48)
Supplement: Additional file 1: Table S1 — Factors considered for the optimization of biosurfactant production under solid state culture (SSC). Table S2. ANOVA analysis of the optimization of production by Nocardiopsis MSA13A. Figure S1. Effect of various substrates including agro-industrial and industrial waste on the production of biosurfactant. The SSC was performed with basal medium 6 ml/250 ml flask, substrate 5 g/250 ml flask and 7 ml/5 g substrate. Figure S2. Effect of various carbon sources on the production of biosurfactant. Figure S3. Effect of various nitrogen sources on the production of biosurfactant. Figure S4. Effect of pH on the production of biosurfactant. Figure S5. Effect of tempertaure on the production of biosurfactant. Figure S6. Effect of incubation period on the production of biosurfactant. Figure S7. Effect of metal ions on the production of biosurfactant. Figure S8. GC-MS data of purified biosurfactant fraction. [file 1472-6750-14-48-S1.doc]

Additional file 1

Table S1. Factors considered for the optimization of biosurfactant production under solid state culture (SSC).

| **Factors** | **Ranges** |
| --- | --- |
| pH | 5 - 9 (with increments of 1) |
| Temperature | 10°C - 50°C (with increments of 10°C) |
| Salinity | 1%, 1.5%, 2%, 2.5%, 3% (with increments of 0.5%) |
| Carbon sources | Glucose, olive oil, kerosene, vegetable oil (1%) |
| Nitrogen source | yeast extract, beef extract, (NH_4_)_2_NO_3_, Acrylamide |
| Metals | FeSO_4_, CuSO_4_, MnCl_2_, MgCl_2_ (1%) |
| Aminoacids | Asparagine, valine, leucine, glycine, glutamicacid (1%) |
| Inoculum size | 1.0, 1.5, 2.0, 2.5, 3.0 ml |
| Incubation time | 5 to 8 d |

Table S2. ANOVA analysis of the optimization of production by *Nocardiopsis* MSA13A.

| **Factors** | **Terms** | **P-value** |
| --- | --- | --- |
| Glucose  Yeast extract  Fe NPs  Glucose  Yeast extract  Fe NPs  Inoculum size | Linear  Linear  Linear  Squared  Squared  Squared  Squared | 0.0003***  0.0094***  0.0002***  0.0006***  0.0403**  0.0238**  0.0006*** |

*-affecting terms, **-Significant, ***- More significant


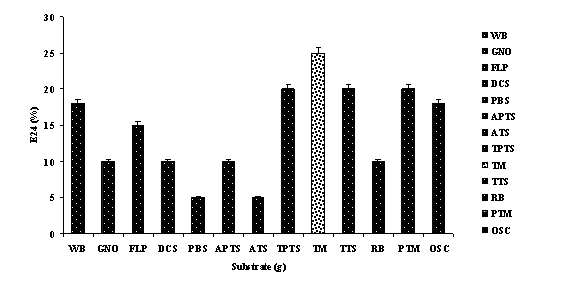


Figure S1. Effect of various substrates including agro-industrial and industrial waste on the production of biosurfactant. The SSC was performed with basal media 6 ml / 250 ml flask, substrate 5 g/250 ml flask and 7 ml / 5 g substrate.

Figure S2. Effect of various carbon sources on the production of biosurfactant

Figure S3. Effect of various nitrogen sources on the production of biosurfactant

Figure S4. Effect of pH on the production of biosurfactant

Figure S5. Effect of tempertaure on the production of biosurfactant

Figure S6. Effect of incubation period on the production of biosurfactant

Figure S7. Effect of metal ions on the production of biosurfactant


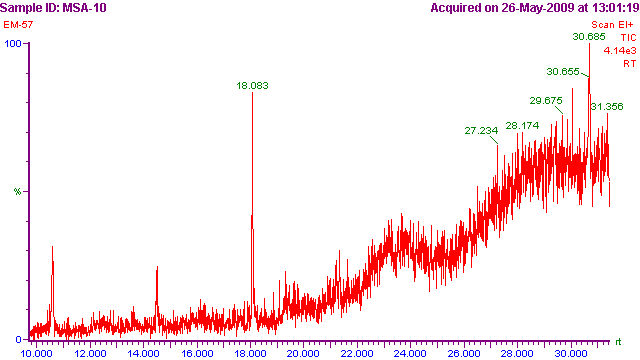


Figure S8 GC-MS data of purified biosurfactant fraction
